# Effect of Artemisinin on the Redox System of NADPH/FNR/Ferredoxin from Malaria Parasites

**DOI:** 10.3390/antiox11020273

**Published:** 2022-01-29

**Authors:** Yoko Kimata-Ariga, Rena Morihisa

**Affiliations:** Department of Biological Chemistry, Graduate School of Sciences and Technology for Innovation, College of Agriculture, Yamaguchi University, Yamaguchi 753-8515, Japan; rena.pon.19980717@gmail.com

**Keywords:** FNR, ferredoxin, artemisinin

## Abstract

FNR and ferredoxin constitute a redox cascade, which provides reducing power in the plastid of malaria parasites. Recently, mutation of ferredoxin (D97Y) was reported to be strongly related to the parasite’s resistance to the front-line antimalarial drug artemisinin. In order to gain insight into the mechanism for the resistance, we studied the effect of dihydroartemisinin (DHA), the active compound of artemisinin, on the redox cascade of NADPH/FNR/ferredoxin in in vitro reconstituted systems. DHA partially inhibited the diaphorase activity of FNR by decreasing the affinity of FNR for NADPH. The activity of the electron transfer from FNR to wild-type or D97Y mutant ferredoxin was not significantly affected by DHA. An in silico docking analysis indicated possible binding of DHA molecule in the binding cavity of 2′5′ADP, a competitive inhibitor for NADPH, on FNR. We previously showed that the D97Y mutant of ferredoxin binds to FNR more strongly than wild-type ferredoxin, and ferredoxin and FNR are generally known to be involved in the oxidative stress response. Thus, these results suggest that ferredoxin is not a direct target of artemisinin, but its mutation may be involved in the protective response against the oxidative stress caused by artemisinin.

## 1. Introduction

**Figure 1:**
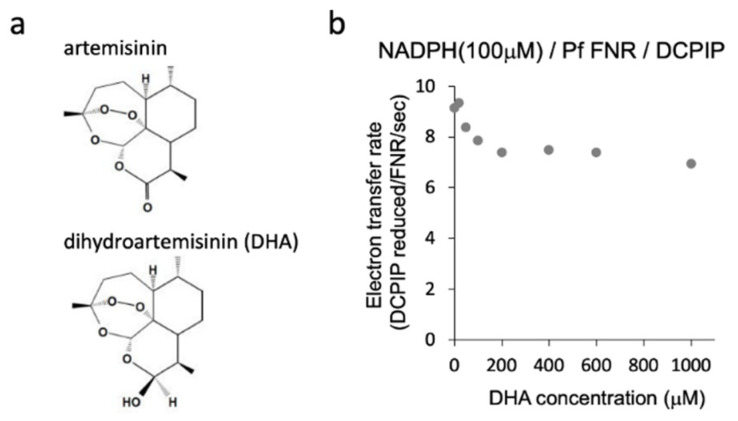
Structure of artemisinin and dihydroartemisinin (DHA) (**a**) and diaphorase activity of PfFNR with different concentrations of DHA (**b**). Diaphorase activity with DCPIP as an electron acceptor was measured in the absence and presence of DHA (at 20 µM–1 mM) under 100 µM of NADPH (non-saturating condition for PfFNR).

Malaria parasite (*Plasmodium* sp.) is a causative agent of malaria. Recently, the emergence of the parasite’s resistance to the frontline antimalarial drug artemisinin (Figure 1a) has become a serious problem. Several genes of the parasite related to the resistance have been found, and among them, the Asp97Tyr mutation of plant type ferredoxin (Fd) in the apicoplast was strongly related to the resistance [1,2].

Apicoplast is a non-photosynthetic plastid organella, which is derived from a red algal symbiont [3] and was shown to be essential for the survival of malaria parasite [4]. The apicoplast supports three metabolic functions: type II fatty acid biosynthesis, heme biosynthesis, and isoprenoid biosynthesis. Isoprenoid biosynthesis was shown to be essential in the erythrocytic stages of the parasite [5]. Fd in plants forms a specific complex with Fd:NADP(H) oxydoreductase (FNR) and constitutes a redox cascade of NADPH/FNR/Fd that provides reducing power to drive the metabolic pathways in plastids. We have shown that Fd and FNR from *Plasmodium falciparum* (PfFd and PfFNR) exhibit the structure and function equivalent to those of the plant counterparts [6] and that the C-terminal region of PfFd, especially the aromatic property of His96, is important for the interaction with PfFNR [7]. Moreover, we showed that the mutation of Asp97Tyr, which is strongly related to the parasite resistance to artemisinin [1,2], significantly increased the affinity (in terms of *K*_m_ and *K*_d_) for PfFNR [7,8] and inhibited the diaphorase activity of PfFNR at a lower concentration compared to wild-type PfFd [8]. 

The action of artemisinin (Figure 1a) in the malaria parasite is thought to involve the cleavage of its endoperoxide bridge and concomitant formation of reactive oxygen species (ROS) and carbon-centered radicals, which would alkylate the essential biomolecules [9,10]. *P. falciparum* is particularly vulnerable to oxidative stress, which may be due to a deficiency of the antioxidant enzymes catalase and glutathione peroxidase. [11]. A close relationship between FNR and oxidative stress has been reported in bacteria [12] and plants [13]. Therefore, it is speculated that PfFNR and its redox partner, PfFd, get involved with the action of artemisinin and, thus, may confer the drug resistance by the mutation of PfFd at Asp97.

In this paper, we studied the effect of the active compound of artemisinin, dihydroartemisinin (DHA; Figure 1a), on the reactions of the redox cascade of NADPH/PfFNR/PfFd using in vitro reconstituted systems. DHA partially inhibited the catalytic activity of PfFNR by decreasing the affinity of PfFNR for NADPH. The activity of the electron transfer from PfFNR to wild-type or D97Y mutant Fd was not significantly affected by DHA. An in silico docking analysis indicated the possible binding of the DHA molecule in the binding cavity of 2′5′ADP, a competitive inhibitor for NADPH, on FNR. The NADPH consumption rate of PfFNR was measured in the absence and presence of various concentrations of PfFd and/or DHA. These results indicated that PfFd is not a direct target of artemisinin, and whether PfFNR is an ‘in vivo’ target for artemisinin is not clear, since the degree of inhibition was small under the current experimental conditions. Instead, PfFd mutation, in combination with PfFNR, may be involved in the protective response against the oxidative stress caused by artemisinin. 

## 2. Materials and Methods

### 2.1. Site-Directed Mutagenesis of PfFd and PfFNR and Preparation of Recombinant Proteins

Cloning and preparation of PfFNR and wild-type and mutant (D97Y) PfFd were described previously [6,7]. The recombinant PfFNR and PfFd proteins were expressed in *Escherichia coli* and purified with the methods described previously [6]. Extinction coefficients of 10 mM^−1^ cm^−1^ at 423 nm and 10 mM^−1^ cm^−1^ at 452 nm [14] were used for the protein concentration determination of PfFd and PfFNR, respectively.

### 2.2. Enzymatic Analyses 

Diaphorase activity of FNR, with DCPIP (2,6-dichlorophenol-indophenol) as an electron acceptor, was measured basically as described previously [15,16]. The reaction mixture contained 1 mM MgCl_2_, 100 mM NaCl, 150 µM DCPIP, 40 nM FNR and NADPH-generating system, 3 mM glucose-6-phosphate, and 30 µg/mL of glucose-6-phosphate dehydrogenase in 50 mM Tris-Cl (pH 7.5). Various concentrations of DHA (D3793; Tokyo Chemical Industry, Tokyo, Japan) were added for the inhibition assay. The reaction was initiated by the addition of NADPH, and the reduction of DCPIP was monitored by the decrease in absorbance at 600 nm at 298 K. The slight reduction of DCPIP observed with the reaction in the absence of FNR was subtracted from the data of each measurement. Steady-state kinetic parameters (*K*_m_ and *V*_max_) were estimated by the fitting of the s–v (substrate concentration vs. measured velocity) plot with a Michaelis–Menten equation using the Solver add-in in Microsoft Excel.

The activity of the NADPH-dependent electron transfer from FNR to Fd was measured using cytochrome *c* (cyt *c*) from horse heart (10,429; Nakalai tesque, Japan) as a final electron acceptor basically as described previously [16]. The reaction mixture contained 100 mM NaCl, 200 µM cyt *c*, 60 nM FNR, 0.5 µM Fd, 400 µM NADPH and NADPH-generating system, 3.0 mM glucose-6-phosphate, and 30 µg/mL of glucose-6-phosphate dehydrogenase in 50 mM Tris-Cl (pH 7.5). The reaction was initiated by the addition of NADPH, and the reduction of cyt *c* was monitored by the increase in absorbance at 550 nm using an extinction coefficient of 21 mM^−1^ cm^−1^ for the reduced minus oxidized form of cyt *c* [17] at 298 K. The slight reduction of cyt *c* observed with the reaction in the absence of Fd was subtracted from the data of each measurement. 

Both diaphorase activity of PfFNR and the electron transfer activity from FNR to Fd (to cyt *c*) described above were measured using a grating microplate reader (model SH-1000Lab, CORONA, Hitachinaka, Japan).

The activity of NADPH oxidation (consumption) in the system containing PfFNR and DHA and/or PfFd was measured in the reaction mixture containing 100 mM NaCl, 100 nM PfFNR, and 165 µM NADPH in 50 mM Tris-Cl (pH 7.5), and DHA and/or Fd was added at various concentrations as shown in the figures. The reaction was initiated by the addition of NADPH, and NADPH oxidation was monitored by the decrease in absorbance at 340 nm at 298 K.

### 2.3. In Silico Docking Analysis 

The 3D structural data of PfFNR (accession code 2OK8, and 2OK7 in complex with 2′5′ADP) and Pea FNR Y308S in complex with NADP^+^ (accession code 1QFZ) were obtained from the Protein Data Bank. The structural data of DHA (ID 456410) was obtained from the PubChem database. 

The ligand docking pose was analyzed through molecular docking experiments. Preparation of the ligand and the docking experiment were carried out using the UCSF Chimera plug-in for AutoDock Vina [18]. The dimension of the gridbox was made so that it covered the whole surface area of PfFNR by dividing it into several parts. The binding energies between PfFNR and the ligands were attained in terms of kcal/mol.

## 3. Results and Discussion

### 3.1. Effect of DHA on the Catalytic Activity of PfFNR

We investigated the effect of DHA on the reduction of PfFNR by NADPH, the first part of the redox cascade involving PfFd and PfFNR, using a diaphorase activity assay with DCPIP as an electron acceptor. Diaphorase activity was measured in the presence of DHA (20 µM–1 mM) under 100 µM of NADPH (non-saturating condition for PfFNR; 110 ± 40 µM of *K*_m_ for NADPH in Table 1 in Ref [16]). As shown in Figure 1b, partial inhibition of the activity by increasing the concentrations of DHA was observed up to 200 µM, and the activity remained constant or slightly decreased by further addition of DHA. The reason for the partial inhibition is not clear at present. The effect by the solvent of DHA, DMSO, was not significant up to 1.8% in the reaction, which corresponds to the addition of DHA at 600 µM, but DMSO may confer a side effect (increase or decrease of the activity) in combination with DHA at higher concentrations. Alternatively, full inhibition by DHA may require its activation (radicalization) by a ferrous iron such as heme and reductant [9,10]. We performed the experiments by the addition of hemin and ascorbate into the assay mixture, but the activity could not be properly measured, probably due to the reduction of the electron acceptor, DCPIP, and changes in the absorption of hemin.

**Figure 2:**
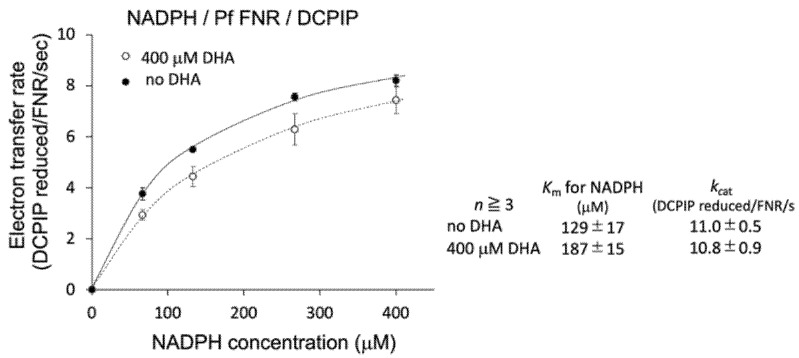
Kinetic analysis of NADPH-dependent diaphorase activity of PfFNR in the absence (closed circles) and presence (400 µM; open circles) of DHA. Each symbol shows the average of the activity, with the error bars representing SD values of at least three measurements. The inlet table shows the average value and SD for the kinetic parameters.

To further investigate the details of this inhibition, the effect of DHA on the kinetic parameters of the reaction was measured. As shown in Figure 2 (inlet table), the *K*_m_ value for NADPH was significantly increased (1.45-fold), while *k*_cat_ was unchanged by the addition of DHA at 400 µM. Therefore, DHA was indicated to decrease the affinity of PfFNR for NADPH. 

### 3.2. Effect of DHA on the Electron Transfer Activity from PfFNR to Wild-Type and Mutant PfFds

**Figure 3:**
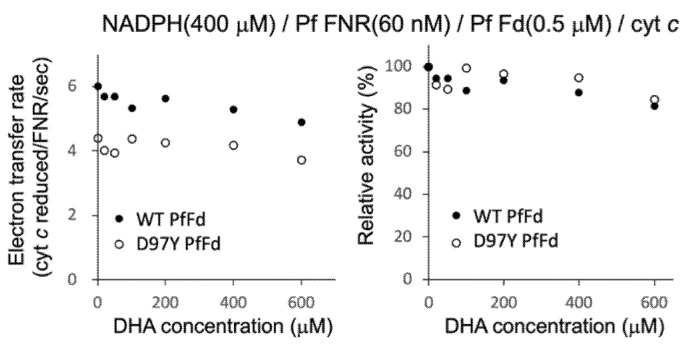
Electron transfer activity from PfFNR to wild-type (closed circles) and D97Y mutant (open circles) PfFds with different concentrations of DHA. The activity using cyt *c* as a final electron acceptor was measured in the absence and presence of DHA (20 µM-600 µM) under 400 µM NADPH and 0.5 µM Fds. The right figure shows the relative activity expressed as a percentage of the electron transfer rate when the rate in the absence of DHA was 100% for each Fd.

Since DHA was shown to affect the affinity of PfFNR for NADPH, next, we investigated the effect of DHA on the electron transfer activity from PfFNR to PfFd (wild-type and D97Y mutant) under a higher concentration of NADPH (400 µM; mostly saturated condition for PfFNR), using cyt *c* as a final electron acceptor (Figure 3). A limited concentration of Fd (0.5 µM) was used, with an aim to investigate the effect of DHA on the affinity of PfFNR for the Fds, but no significant inhibition was observed for both wild-type and mutant Fds up to 400 µM of DHA. Furthermore, no significant effect of DHA (at 400 µM) on *K*_m_ and *k*_cat_ was observed for both Fds (data not shown). As mentioned in the previous section, the activation of DHA may be required for the inhibition. However, the addition of hemin and reductant into this assay system would be expected to cause an inaccurate measurement under the current experimental conditions. Therefore, the effect of DHA (up to 400 µM) on the electron transfer from PfFNR to PfFd (wild-type and D97Y mutant) was thought to be non-significant based on the results obtained under the current experimental conditions.

### 3.3. Reaction of PfFNR with DHA

**Figure 4:**
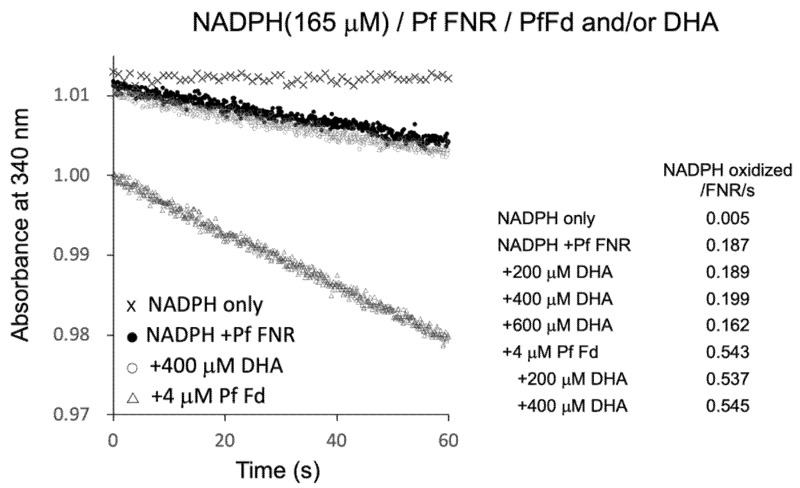
NADPH oxidation by PfFNR (closed circles), PfFNR plus DHA (open circles), and/or PfFd (triangles). The activity was measured under 165 µM NADPH with different concentrations of DHA in the absence and presence of Fd at 4 µM. Absorption at 340 mm was monitored at 0.1 s intervals except for the measurement of ‘NADPH only‘ at 1 s intervals.

PfFNR was shown to reduce a series of redox cycling xenobiotics, such as quinones and nitroaromatic compounds, and was considered as a possible source of their radical formation in *P. falciparum* [19,20,21]. Then, the electron transfer reaction of PfFNR in the presence of DHA was investigated by monitoring NADPH consumption as was used in the study to detect the reduction of xenobiotics [21]. As shown in Figure 4, NADPH was oxidized by PfFNR (closed circles) at a rate of 0.187 NADPH oxidation/FNR/sec., which was thought to represent the electron transfer from FNR to oxygen in the reaction solution. The addition of Fd at 4 µM (open triangles) increased the NADPH oxidation by about 3 times, which was thought to represent the rate of the electron transfer from Fd to oxygen (cf. one-tenth of the rate of ascorbate radical reduction and one-third of the rate of electron transfer to heterologous Fd-dependent nitrite reductase in a similar system in Ref. [8]). On the other hand, the addition of DHA (200–400 µM) did not appear to increase the rate of NADPH oxidation significantly (open circles at 400 µM DHA, and inlet table), but it even decreased at higher concentration (600 µM).

The radical formation of artemisinin was indicated to occur by Fenton reaction in the presence of heme (Fe^2+^) derived from hemoglobin in vivo [22]. Thus, to investigate the possible role of Fd as the source of Fe^2+^ for radical formation of DHA, Fd and DHA were added to the reaction, but there was no significant increase in the NADPH oxidation as compared to that in the absence of DHA (bottom three of inlet table in Figure 4). Therefore, the reduced Fd may not function as a source of a Fenton reaction for the radical formation of DHA. Since the radical formation of xenobiotics by PfFNR was measured by cyt *c* reduction in the report by Lesanavičius, M. et.al. [21], we also measured the cyt *c* reduction using the same system, but there was no significant increase in the cyt *c* reduction by addition of DHA (data not shown). These results did not provide evidence for the electron transfer from PfFNR to DHA and for the radical formation of DHA under the current experimental conditions.

**Figure 5:**
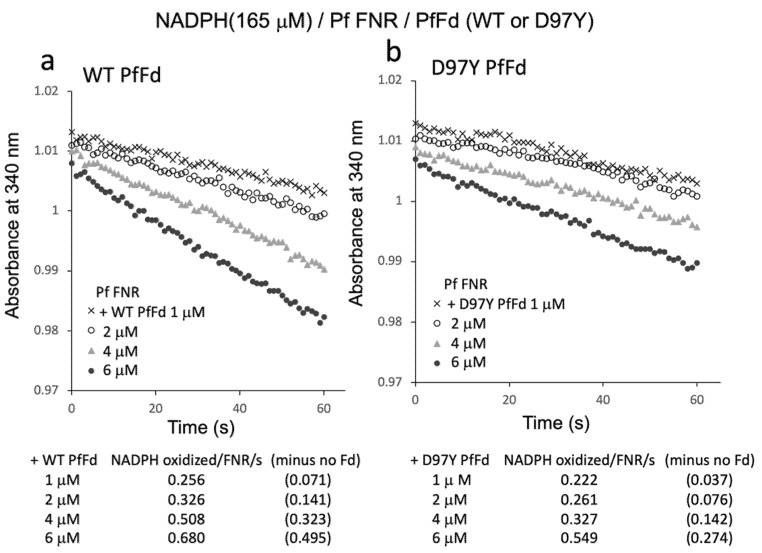
NADPH oxidation by PfFNR with different concentrations of wild-type (**a**) or D97Y PfFd (**b**). The activity was measured under 165 µM NADPH. The values in parentheses are the values after subtracting the activity in the absence of Fd (0.185 NADPH oxidized/FNR/s).

Since the results above indicated the electron transfer activity from PfFNR/PfFd to oxygen in the reaction solution, the Fd-concentration dependency of this activity was investigated (Figure 5). Fd-concentration-dependent NADPH oxidation was observed for both wild-type PfFd and D97Y PfFd, but the activity of the D97Y mutant was roughly half of that of the wild-type PfFd, of which the tendency was similar to the previous results of the electron transfer activity from PfFd and D97Y PfFd (at 5 µM) to a heterologous Fd-dependent enzyme, cyanobacterial nitrite reductase (1.45 and 0.62 NADPH oxidation/FNR/sec., respectively, in Ref. [8]). 

### 3.4. In Silico Docking Analysis of PfFNR and DHA 

**Figure 6:**
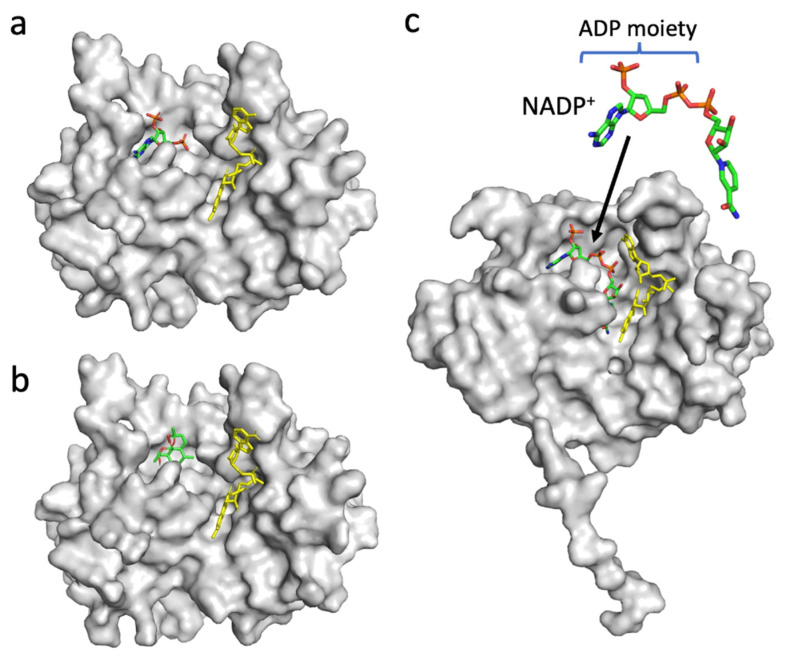
Structures of PfFNR complexed with 2′5′ADP (accession code 2OK7) (**a**), PfFNR docked with DHA in silico (**b**), and Pea FNR (Tyr308Ser) mutant complexed with NADP^+^ (accession code 1QFZ) (**c**). The FAD moiety is shown as a yellow-colored stick model (**a**–**c**), and 2′5′ADP (**a**), DHA (**b**), and NADP^+^ (**c**) are shown as stick models. (**b**) The docking structure obtained with the lowest binding energy (ΔG) is shown.

Since the decrease in the affinity between PfFNR and NADPH by DHA was indicated by a diaphorase assay (Figure 2), the binding of DHA on PfFNR was investigated by an in silico docking analysis. Although the 3D structure of the complex of PfFNR with NADP(H) was not available, the structure of the complex of PfFNR with an NADP(H) analog, 2′5′ADP (FNR inhibitor), (accession code 2OK7) was reported (Figure 6a) [14]. The docking analysis of 2′5′ADP with PfFNR resulted in a structure with an almost identical binding site and mode of 2′5′ADP on PfFNR as those of the reported structure of PfFNR-2′5′ADP (Figure S1). The calculated binding energy (ΔG) was −8.0 kcal/mol, with the structure of the highest score. Then, a docking analysis of DHA and PfFNR resulted in the structures with several conformers of DHA (Figure S2), but the structure with the lowest calculated binding energy (−7.0 kcal/mol) was that with DHA bound in the same cavity as 2′5′ADP (Figure 6a,b). The structure was also compared with that of the Pea FNR Y308S:NADP^+^ complex (Figure 6c), since the PfFNR:NADP(H) complex and other FNR:NADP(H) complexes from plants have not been obtained; Pea FNRY308, of which the C-terminal Tyr is substituted by Ser, has a much higher affinity for NADP(H) [23]. The ADP moiety of NADP^+^ (as shown in Figure 6c) binds to the cavity, which corresponds to those of DHA and 2′5′ADP binding in PfFNR (Figure 6a,b), indicating that DHA could compete with NADPH for binding to PfFNR and, thus, could reduce the affinity of PfFNR for NADP(H) as seen in the kinetic results (Figure 2).

To sum up, DHA was shown to partially inhibit the activity of PfFNR by decreasing the affinity for NADPH, and its possible binding into the binding cavity of ADP moiety of NADP(H) on PfFNR was suggested by the docking analysis. Evidence for the direct effect of DHA on Fd function (binding or electron transfer to PfFNR) was not obtained. 

These results indicate that PfFd is not a direct target of artemisinin, and whether PfFNR is an ‘in vivo’ target for artemisinin is not clear, since the degree of inhibition was small under the current experimental conditions. Instead, PfFd mutation in combination with PfFNR may modulate the protective response against the oxidative stress caused by artemisinin.

Concerning the ‘in vivo’ action of artemisinin, artemisinin is thought to be activated (radicalized) primarily by heme, which is mainly derived from hemoglobin digestion in the food vacuole of malaria parasites in red blood cells [10]. Activated artemisinin and the resulting reactive oxygen species (ROS) can react promiscuously with a wide range of cellular targets, disrupting cellular protein homeostasis [22,24]. Several mutations associated with artemisinin resistance were reported in the *P. falciparum* genes, including PfFd and ribosomal protein S10 in apicoplasts, autophagy-related protein, purine nucleoside phosphorylase, and chloroquine resistance transporter. Among them, the mutations of the kelch family protein K13 (assumed to be either a regulator of polyubiquitination of PfPI3K or a ubiquitin E3 ligase substrate adaptor) are considered as the key determinant of artemisinin resistance [24]. The molecular mechanisms of K13-mediated artemisinin resistance involve reduced hemoglobin uptake/digestion and an increased cellular stress response against ROS, in which K13 mutations alter multiple aspects of the parasite’s intra-erythrocytic developmental program. These changes impact cell-cycle periodicity, the unfolded protein response, protein degradation, vesicular trafficking, and mitochondrial metabolism [24].

In this connection, photosynthetic electron transport is a source of superoxide, and FNR together with Fd is known to play a central role in the chloroplast oxidative stress response [13]. The majority of Fd reduced by photosystem I is oxidized by FNR to reduce NADP^+^ to NADPH, and several other enzymes are known to be involved; the superoxide radical is removed in the water–water cycle by the action of super oxide dismutase and ascorbate peroxidase, resulting in oxidation of ascorbate to dehydroascorbate [25]. Reductive regeneration of ascorbate can be supported directly by Fd or through glutathione (GSH) oxidation to GSSG by dehydroascorbate reductase. GSH regeneration is supported in turn by NADPH, through the action of glutathione reductase. Thus, both Fd and FNR are involved in the removal of ROS. NADPH-dependent thioredoxin reductase C supports antioxidant metabolism by regenerating 2-Cys peroxiredoxin [26]. Fd and FNR in malaria parasites may have a similar function although the presence of the related enzymes has not been well investigated yet. Thus, PfFd together with PfFNR may be involved in the protective response against the oxidative stress caused by artemisinin, instead of being a direct target of artemisinin, as well as in the case of the K13-related response; the D97Y mutant of PfFd, which exhibits a higher affinity for PfFNR and possibly different redox property, may reinforce the above function.

In addition, PfFd mutation may also modulate PfFd-dependent metabolism. For example, isoprenoid biosynthesis in the apicoplast was shown to be involved in a major process that is critical for the parasite tolerance to febrile temperature and artemisinin [27]. The major function of isoprenoid biosynthesis is in protein-prenylation that regulates protein-targeting, including vesicular trafficking of unfolded proteins during the stress response. Because enzymes (IspG and IspH) in this pathway of *Plasmodium* sp. are known to be Fd-dependent [28,29], the a mutation of PfFd may affect the isoprenoid biosynthesis, leading to the tolerance to artemisinin. 

These possibilities need to be investigated.

## 4. Conclusions

We studied the effect of antimalarial drug artemisinin (DHA) on the redox cascade of NADPH/FNR/Fd from malaria parasites. DHA partially inhibited the diaphorase activity of FNR by decreasing the affinity of FNR for NADPH. The activity of the electron transfer from FNR to wild-type or D97Y mutant Fd was not significantly affected by DHA. An in silico docking analysis indicated possible binding of the DHA molecule in the binding cavity of 2′5′ADP, a competitive inhibitor for NADPH, on FNR. These results indicate that Fd is not a direct target of artemisinin, and whether FNR is an ‘in vivo’ target for artemisinin is not clear, since the degree of inhibition was small under the current experimental conditions. Instead, Fd mutation in combination with FNR may modulate the protective response against the oxidative stress caused by artemisinin. 

## Data Availability

Data is contained within the article.

## Supplementary Materials

The following are available online at https://www.mdpi.com/article/10.3390/antiox11020273/s1, Figure S1: Overlay of the structures of PfFNR complexed with 2′5′ADP (accession code 2OK7) and PfFNR docked with 2′5′ADP in silico. Figure S2: Overlay of the conformers of DHA docked on PfFNR in silico.
