# Supplementary material for: Effect of Artemisinin on the Redox System of NADPH/FNR/Ferredoxin from Malaria Parasites
Source: Antioxidants (Basel). 2022 Jan 29;11(2):273. doi: 10.3390/antiox11020273 (PMC8868210; doi:10.3390/antiox11020273)
Supplement: Supplementary file 1 [file antioxidants-11-00273-s001.zip › antioxidants-1520819-supplementary.pdf]

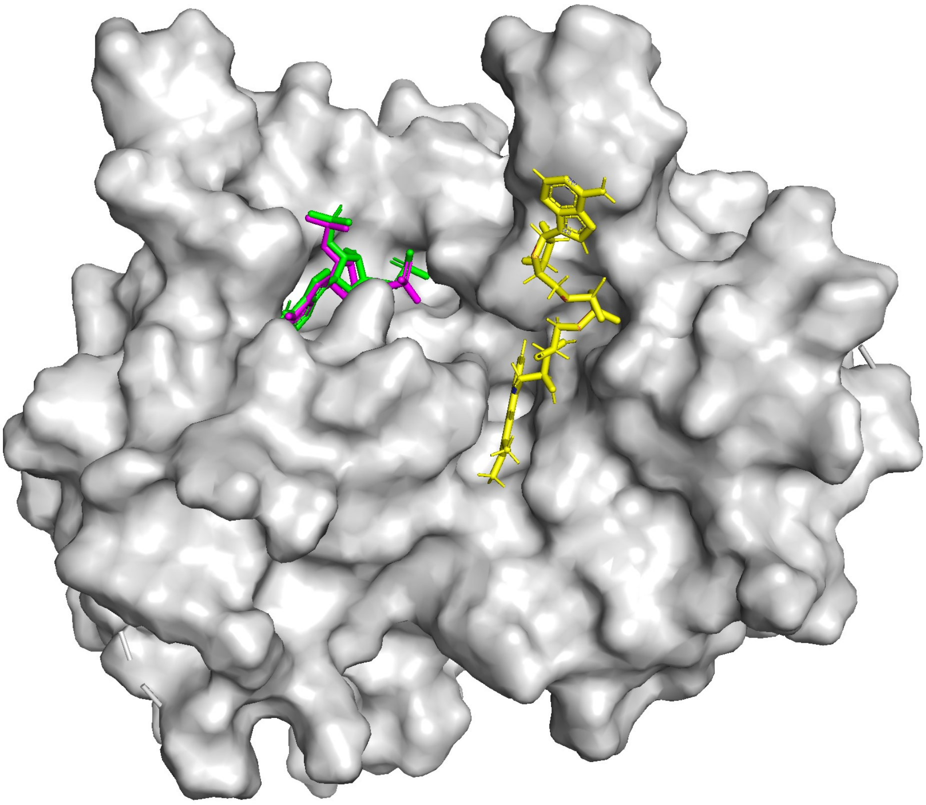

Figure S1. Overlay of the structures of PfFNR complexed with 2'5'ADP (accession code 2OK7) and PfFNR docked with 2'5'ADP *in silico.* FAD moiety is shown as yellow-colored stick model, and 2'5'ADP in complex structure (pink) and in docked structure (green) are shown as stick model. The docking structure obtained with the lowest binding energy (ΔG) is shown.

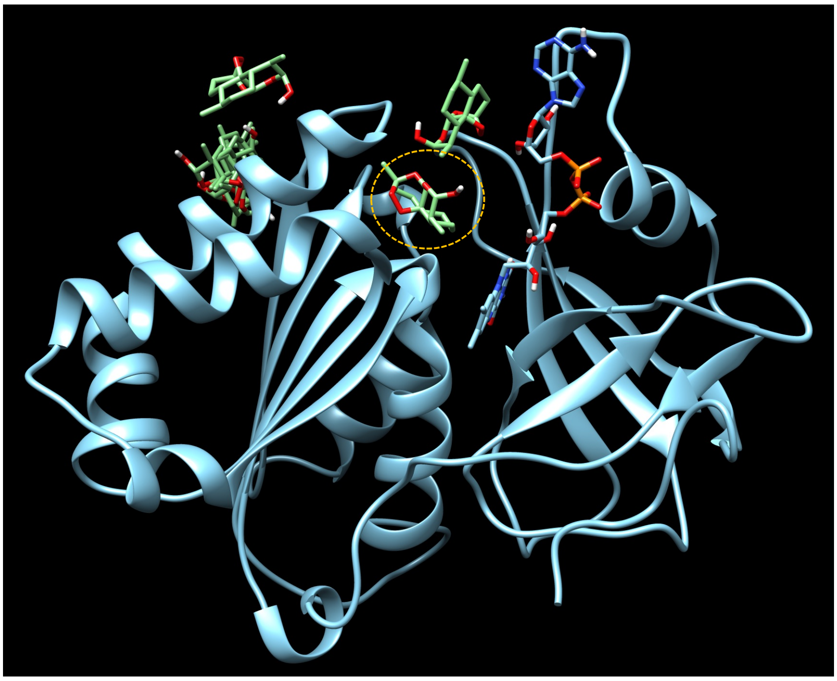

Figure S2. Overlay of the conformers of DHA docked on PfFNR *in silico.* FAD moiety (pale blue) and DHA (green) are shown as stick model. The DHA conformer obtained with the lowest binding energy (ΔG) is shown in a dotted circle.
